# Photochemical Efficiency of Photosystem II in Inverted Leaves of Soybean [*Glycine max* (L.) Merr.] Affected by Elevated Temperature and High Light

**DOI:** 10.3389/fpls.2021.772644

**Published:** 2022-02-16

**Authors:** Cong Wang, Qiuli Gu, Lianjia Zhao, Chunyan Li, Jintao Ren, Jianxin Zhang

**Affiliations:** ^1^College of Agriculture, Xinjiang Agricultural University, Urumqi, China; ^2^Agriculture and Rural Bureau of Qapqal County, Qapqal County, China; ^3^Research Institute of Crop Germplasm Resources, Xinjiang Academy of Agricultural Sciences, Urumqi, China

**Keywords:** high light, elevated temperature, leaf inversion, photosynthesis, chlorophyll *a* fluorescence

## Abstract

In summer, high light and elevated temperature are the most common abiotic stresses. The frequent occurrence of monsoon exposes the abaxial surface of soybean [*Glycine max* (L.) Merr.] leaves to direct solar radiation, resulting in irreversible damage to plant photosynthesis. In this study, chlorophyll *a* fluorescence was used to evaluate the functional status of photosystem II (PSII) in inverted leaves under elevated temperature and high light. In two consecutive growing seasons, we tested the fluorescence and gas exchange parameters of soybean leaves for 10 days and 15 days (5 days after recovery). Inverted leaves had lower tolerance compared to normal leaves and exhibited lower photosynthetic performance, quantum yield, and electron transport efficiency under combined elevated temperature and high light stress, along with a significant increase in absorption flux per reaction center (RC) and the energy dissipation of the RC, resulting in significantly lower performance indexes (PI_ABS_ and PI_total_) and net photosynthetic rate (P_*n*_) in inverted leaves. High light and elevated temperature caused irreversible membrane damage in inverted leaves, as photosynthetic performance parameters (P_*n*_, PI_ABS_, and PI_total_) did not return to control levels after inverted leaves recovered. In conclusion, inverted leaves exhibited lower photosynthetic performance and PSII activity under elevated temperature and high light stress compared to normal leaves.

## Introduction

Soybean leaves are heterogeneous, and the adaxial surface is the major contributor to carbon gain because the adaxial surface palisade tissue is rich in chloroplasts and exposed to direct radiation (Evans, 1999). However, some plant leaves are inverted or wobbly due to cultivation conditions (e.g., water and fertilizer) and wind (Zhang et al., 2016; Paradiso et al., 2020). Due to the difference in anatomy between the abaxial and adaxial surfaces of soybeans, their response to environmental conditions can vary, especially light conditions (Hughes and Smith, 2007). Therefore, studying the response of inverted leaves to the environment will provide a theoretical basis for exploring ways to minimize damage to the photosynthetic apparatus.

Soybean is one of the most important oil crops in the world (Cai et al., 2020); due to human factors and frequent natural disasters, the global average temperature will continue to rise rapidly in the future; and unfavorable high temperatures will affect plant growth and development (IPCC, 2019), usually causing reversible/irreversible damage to different organs of the plant, this is because leaf photosynthesis is one of the most sensitive processes to elevated temperatures in plants (Yamori and Shikanai, 2016; Mihaljević et al., 2020). Under natural conditions, the elevated temperatures at noon in summer are usually accompanied by other environmental stresses, such as high light, and the dual stress of heat and high light seriously affects the growth and development of soybeans, especially during the seed-filling stage, resulting in reduced soybean yields (Cohen et al., 2021; Kimm et al., 2021). As an important organ in direct contact with the environment, leaves are more sensitive to light and temperature, because photosystem II (PSII) is sensitive to heat and high irradiation stress during the process of carbon dioxide assimilation (Dongsansuk et al., 2013; Jiang et al., 2021). Exposure to high light and elevated temperature in summer can damage the photosynthetic apparatus of the plant and cause photoinhibition, which is manifested in the metabolic processes: reduced transpiration accompanied by increased leaf temperature, reduced antioxidant and photosynthetic enzyme activities, damage to the cytoplasmic membrane, destruction of chloroplast structure and function, reduced electron transport and carbon metabolism, and increased reactive oxygen species (ROS) (Janka et al., 2013; Gu et al., 2017; Blackhall et al., 2020; Mihaljević et al., 2020). Studies have found that the synergistic effect of elevated temperature and high light caused significant degradation of D1 protein in plants, causing damage to both the donor and acceptor side of PSII (Krieger-Liszkay et al., 2008; Kalaji et al., 2016). Sunburn occurs on leaves under the long-term high light, and sunspots were also found on some fruits, which seriously affects fruit quality and crop yield (Chen et al., 2012; Blackhall et al., 2020). Under natural conditions, heat and high light stress often occur simultaneously and tend to damage the photosynthetic apparatus of inverted leaves; nevertheless, the state of the photosynthetic system of inverted leaves under elevated temperature and high irradiation needs further study.

The rapid chlorophyll *a* fluorescence technique is a nondestructive and effective tool for monitoring the effects of abiotic stress on the photochemical efficiency of PSII and the health of the plant, because it quickly, noninvasively analyzes and provides powerful data related to photosynthesis (Strasser and Srivastava, 1995; Oukarroum et al., 2018). The typical rise in chlorophyll *a* fluorescence transient kinetics over 1 s is multiphase (OJIP curve), and the shape of the OJIP curve changes with the physiological condition of the plant, reflecting valuable information on the structure and function of the photosynthetic apparatus (Kalaji et al., 2016). Strasser et al. (2004) developed a data processing method (JIP-test) for rapid chlorophyll *a* fluorescence induction curves based on the theory of energy fluxes in thylakoid membranes. The specific flux of each reaction center (RC) and the apparent flux of excited leaf cross-section (CS_*O*_) provide rich information about the redox state of PSII (Strasser et al., 2010; Kalaji et al., 2016). The JIP-test has been widely used to analyze crop tolerance to single abiotic stresses and to screen for indicators of resistance identification, for example, elevated temperature stress (Janka et al., 2013; Mihaljević et al., 2020), nutrient deficiencies (Kalaji et al., 2014), drought stress (Marcińska et al., 2017), and high light stress (Hazrati et al., 2016).

Previous studies have shown that the photochemical efficiency of leaves is reduced when the leaf is inverted (Paradiso et al., 2020). In this study, we aimed to investigate the daily response of inverted leaves under specific conditions of high temperature and high irradiation at the photosynthetic level. We used JIP-test and gas exchange parameters to evaluate the photochemical adaptation of inverted leaves under elevated temperature and high light at noon. We hypothesized that PSII function is weaker and photochemical efficiency is lower in inverted leaves under high temperature and high light compared to normal leaves.

## Materials and Methods

### Experimental Field and Meteorological Conditions

**FIGURE 1:**
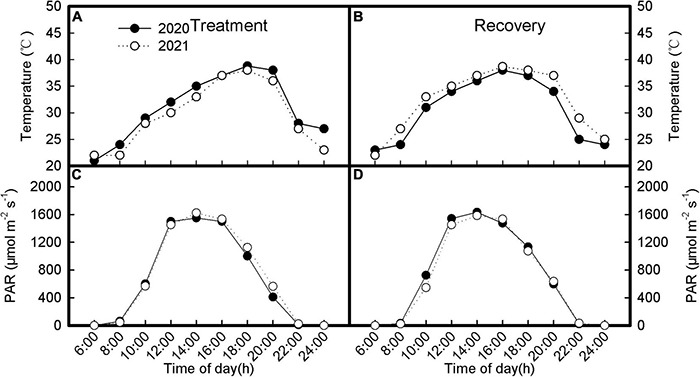
Meteorological data during treatment **(A,C)** and recovery **(B,D)** when measuring gas exchange and chlorophyll *a* fluorescence.

The field experiment was carried out in the Yili Institute of Agricultural Science in Xinjiang Production, China (43°50′N, 80°04′E). The experimental field was clay loam soil. The physicochemical properties of soil at 0–20 cm soil layer were as follows: available N 51.3 mg/kg, available P 15.8 mg/kg, and available K 102.1 mg/kg. For a better understanding of obtained results about the acclimatization of the photosynthetic apparatus of inverted leaves to heat and high light, we showed the data of air temperature and solar radiation measured on the day after treatment and recovery. The meteorological data were obtained from artificial weather devices placed in the test field as shown in Figures 1A–D. When the photosynthetic capacity of soybean leaves is measured, the temperature at 4 p.m. in summer is 38.2°C, and the light intensity is 1,512 μmol(CO_2_) m^–2^ s^–1^, which is much higher than its light saturation point.

### Experimental Design

Spring soybean Heinong 87 from the Heilongjiang Academy of Agricultural Sciences of China (45° 58 N, 126° 48 E) was used as the experimental material. The experimental treatments consisted of leaf inversion treatment that the small middle leaves of four sections from the top of the plant were fixed with fine cotton thread and made to face up on the abaxial leaf surface during the seed-filling stage, then return to the original shape 15 days after the treatment, and do nothing for the control treatment. The experiment was conducted in a completely randomized block design and repeated three times. The plots were 4 m × 10 m. The soybean cultivar was sown with a density of 25.0 plants/m^2^ on April 15, 2020 and 2021, the row spacing of 40 cm, and the plant spacing of 10 cm. Other management referred to local high-yield practices.

### Measurement of Photosynthetic Traits

We tried to choose the days with high temperature and solar radiation for measurement. The adaxial surface of fully expanded leaves in the main stem for both treatments was illuminated when they were inside the CIRAS chamber in both treatments. The net photosynthetic rate (P_*n*_), transpiration rate (T_*r*_), stomatal conductance (gs), and intercellular carbon dioxide concentration (C_*i*_) were measured from both treatments at 10 and 15 days (recovery) after leaf treatments, in the morning (9 a.m.) and afternoon (4 p.m.) using a portable photosynthesis system (CIRAS-3, PP Systems, London, United Kingdom). Steady-state photosynthesis was achieved after the leaves were clamped for 5 min, and the photosynthetic parameters were recorded at 1,800 μmol m^–2^ s^–1^ light intensity, 400 ± 5 μmol mol**^–^**^1^ CO_2_, and 70% humidity.

### Photosynthetic Light-Response Curves

Photosynthetic light-response curves of fully expanded leaves in the main stem of soybean were measured 10 days after leaf inversion using a portable photosynthesis system (CIRAS-3, PP Systems, London, United Kingdom) between 11:00 a.m. and 1:30 p.m. at the soybean R5 expanding stage. The P_*N*_ was recorded at photosynthetic photon flux densities (PPFDs) of the following: 2,000; 1,800; 1,500; 1,200; 1,000; 800; 600; 400; 200; 150; 100; 50; 30; and 0 μmol m**^–^**^2^ s**^–^**^1^, respectively. These measurements were recorded at a fixed CO_2_ concentration of 400 ± 5 μmol mol**^–^**^1^ using CO_2_ cylinders. The photosynthetic light-response curves can be fitted with a nonlinear hyperbolic model (Farquhar et al., 1980) as follows:


$$P_{\text{N}}\left(\text{I}\right)=\frac{\alpha \text{I}+P_{\text{Nmax}}-\sqrt{\left(\alpha \text{I}+P_{\text{N}\max}\right)\alpha \text{I}+P_{\text{N}\max}-4\alpha \text{I}P_{\text{N}\max}}}{2\theta }-R_{D}$$

where α is the apparent quantum yield (AQY), I represents the PPFD, P_*Nmax*_ is the maximum net photosynthetic rate, R_*D*_ is the dark respiration rate, and θ is the convexity. The linear regression analysis was performed using SPSS version 19.0 software (IBM, Chicago, IL, United States) in the PPFD of 0–2,000 μmol m**^–^**^2^ s**^–^**^1^. The crossover point of this line with the x-axis (photosynthetically active radiation, PAR) was the light compensation point (LCP, μmol m**^–^**^2^ s**^–^**^1^), whereas the corresponding x-axis value for the crossover points along the y-axis was the light saturation point (LSP, μmol m**^–^**^2^ s**^–^**^1^).

### Chlorophyll *a* Fluorescence

The rapid chlorophyll *a* fluorescence induction kinetics were measured using a Plant Efficiency Analyzer (Handy-PEA, Hansatech, Norfolk, United Kingdom) at 9 a.m. and 4 p.m. 10 and 15 days after treatment. The leaves (from 10 individual plants) per treatment were dark-adapted using a fixing leaf clip (Hansatech) for 30 min. The samples were illuminated with 660-nm light of 3,000 photons μmol m^–2^ s^–1^ for 1 s, and all the collected data were analyzed using the program plant efficiency analyser (PEA) Plus to obtain OJIP-test parameters (Kalaji et al., 2012), as shown in Table 1. To further analyze the difference in fluorescence kinetics between morning and afternoon measurements in response to elevated temperature and excess light, the original chlorophyll *a* fluorescence (OJIP) transients were normalized between minimum fluorescence when all PSII RCs were open (F_*O*_) and maximum fluorescence when all PSII RCs were closed (F_*m*_): the relative variable fluorescence was expressed as V_*OP*_ [V_*OP*_ = (F_*t*_−F_*O*_)/(F_*m*_−F_*O*_)], and the difference between the transients was expressed as △V_*OP*_ [△V_*OP*_ = V_*OP*_(measurement at 4 p.m.)-V_*OP*_(measurement at 9 a.m.)]. The original OJIP transients that were normalized between F_*O*_ and F_*K*_ were expressed as V_*OK*_ [V_*OK*_ = (F_*t*_−F_*O*_)/(F_*K*_−F_*O*_)], between F_*O*_ and F_*J*_ were expressed as V_*OJ*_ [V_*OJ*_ = (F_*t*_−F_*O*_)/(F_*J*_−F_*O*_)], between F_*J*_ and F_*I*_ were expressed as V_*JI*_ [V_*JI*_ = (F_*t*_−F_*J*_)/(F_*I*_−F_*J*_)], and between F_*I*_ and F_*P*_ were expressed as V_*IP*_ [V_*IP*_ = (F_*t*_−F_*I*_)/(F_*P*_−F_*I*_)]; finally, the differences between the transients which were expressed as △V_*OK*_ [△V_*OK*_ = V_*OK*_(measurement at 4 p.m.)-V_*OK*_(measurement at 9 a.m.)], △V_*OJ*_ [△V_*OJ*_ = V_*OJ*_(measurement at 4 p.m.)-V_*OJ*_(measurement at 9 a.m.)], △V_*JI*_ [△V_*JI*_ = V_*JI*_(measurement at 4 p.m.)-V_*JI*_(measurement at 9 a.m.)], △V_*IP*_ [△V_*IP*_ = V_*IP*_(measurement at 4 p.m.)-V_*IP*_(measurement at 9 a.m.)], and △V_*OP*_ [△V_*OP*_ = V_*OP*_(measurement at 4 p.m.)-V_*OP*_(measurement at 9 a.m.)] were determined for visualization (Li et al., 2020).

**TABLE 1:** Kinetic parameters of chlorophyll fluorescence.

	Fluorescence parameter	Description
Extracted parameter	V_*K*_=(F_300μ *s*_−F_*O*_)/(Fm-F_*O*_)	Relative variable fluorescence at 300 μs after illumination of a dark-adapted sample
	V_*J*_=(F_2*ms*_-F_*O*_)/(Fm-F_*O*_)	Relative variable fluorescence at 2 ms after illumination of a dark-adapted sample
	V_*I*_=(F_30*ms*_-F_*O*_)/(Fm-F_*O*_)	Relative variable fluorescence at 30 ms after illumination of a dark-adapted sample
	V_*K*_/V_*J*_	Limitation/inactivation and possibly damage of the oxygen-evolving complex
	Area	Density area over the chlorophyll a fluorescence transient delimited by a horizontal line at F_*m*_
	F_*O*_	Minimum fluorescence, when all PS II reaction center (RC) was open
	F_*m*_	Maximum fluorescence, when all PS II RC was closed
	F_*V*_ = F_*m*_-F_*O*_	Maximum variable fluorescence
	Mo=4(F_300μ *s*_-F_*O*_)/(Fm-F_*O*_)	Approximated initial slope of the fluorescent transient. This parameter is related to rate of closure of reaction centers
	F_*V*_/F_*O*_	maximum ratio of quantum yields of photochemical and concurrent non-photochemical processes in PS II
Specific fluxes per RC	RC/CS=Fo×φ_*PO*_×V_*J*_/Mo	Density of active RCs (Q_*A*_ reducing RCs) per cross section at point 0
	ABS/RC=M_*O*_×(1/V_*J*_)×[1-(F_*O*_/Fm)]	Absorption flux per RC
	DI_*O*_/RC=(ABS/RC)-(TR_*O*_/RC)	Dissipated energy flux per RC
	TR_*O*_/RC=M_*O*_×(1/V_*J*_)	Trapped energy flux per RC
	ET_*O*_/RC=M_*O*_×(1/V_*J*_)×Ψ_*EO*_	Electron transport flux per RC
	RE_*O*_/RC=(ET_*O*_/RC)×δ_*RO*_	Reduction of end acceptors at PS I electron acceptor side per RC
Yield or flux ratio	φ_*PO*_=F_*v*_/F_*m*_	Maximum quantum yield of PSII photochemistry
	Ψ_*EO*_=ET_*O*_/TR_*O*_=1-V_*J*_	Probability that a trapped exciton moves an electron into the trapped electron transport chain beyond Q_*A*_^–^
	φ_*EO*_=(F_*v*_/F_*m*_) (1-V_*J*_)	Quantum yield for electron transport at t = 0
	δ_*RO*_=(1-V_*I*_) (1-V_*J*_)	Efficiency with which an electron can move from the reduced intersystem electron acceptors to the PS I end electron acceptors
	φ_*RO*_ = φ_*PO*_×Ψ_*EO*_×δ_*RO*_	Quantum yield for the reduction of end acceptors of PS II per photon absorbed
Performance index (PI)	PI_ABS_=(RC/ABS)[φ_*PO*_/(1-φ_*PO*_)][Ψ_*EO*_ /(1-Ψ_*EO*_)]	PI on absorption basis
	PI_total_=PI_ABS_×δ_*RO*_/(1-δ_*RO*_)	Total PI, measuring the performance up to the PS I end electron acceptors

### Data Analysis

The PEA Plus software was used to obtain the OJIP-test parameters. The differences between data at the two measurement time points (morning and afternoon) and between leaf treatments were analyzed by one-way ANOVA with the SPSS version 19.0 (SPSS Inc., Chicago, IL, United States). The structures of variability and correlations between the measured parameters were explored by the principal component analysis (PCA), the selection of the principal factors was based on those with eigenvalues greater than 1. The data are presented as the mean ± SE, and the means were compared using least significant difference (LSD) tests, *P < 0.05 and ^**^P < 0.01. The graphs were constructed using SigmaPlot software, version 12.5.

## Results

### Leaf Gas Exchange

**FIGURE 2:**
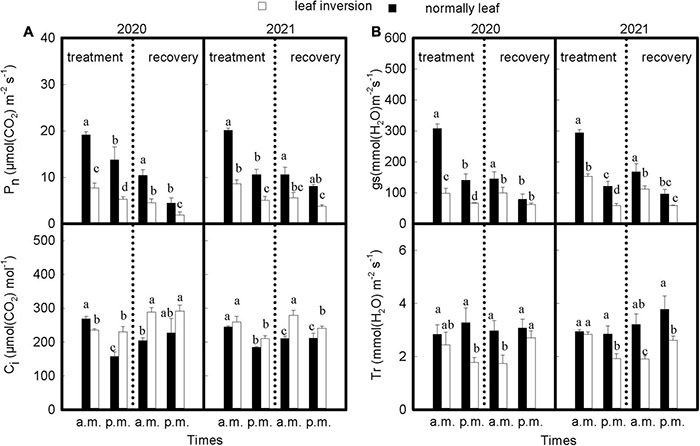
**(A,B)** The gas exchange parameters of both treatments during 2020 and 2021 at 9 a.m. and 4 p.m. Data are expressed as means ± SEs (*n* = 3). P_*n*_, net photosynthetic rate; C_*i*_, intercellular CO_2_ concentration; gs, stomatal conductance; T_*r*_, transpiration rate. Different letters represent significant differences (*P* < 0.05) between treatment and time of measurement. The vertical dotted line separates treatment from recovery.

As shown in Figures 2A,B, the P_*n*_, gs, and C_*i*_ values of normal and inverted leaves (except C_*i*_ values for inverted leaves in 2020) were significantly reduced at noon during treatment compared with those measured in the morning (*P* < 0.05), and the decline was higher for inverted leaves than for normal leaves; T_*r*_ values for inverted leaves were significantly lower (*P* < 0.05) than for normal leaves at noon measurements, and the T_*r*_ values of the normal leaves was not significantly different between 9 a.m. and 4 p.m. (*P* > 0.05), while inverted leaves decreased significantly at 4 p.m. compared to the 9 a.m. measurement (*P* < 0.05). After recovery of inverted leaves, the P_*n*_ and gs values of all treated leaves showed the same trend as leaves during treatment; C_*i*_ and T_*r*_ values of normal and inverted leaves were elevated at noon compared to morning measurements, with higher C_*i*_ values of inverted leaves than normal leaves, but the opposite for T_*r*_ values.

### Photosynthetic Light-Response Curves

The ANOVA showed that P_*Nmax*_, AQY, R_*D*_, LCP, and LSP were affected by leaf inversion (*P* < 0.05) when the leaf was inverted and restored (Table 2). During leaf inversion, P_*Nmax*_, AQY, R_*D*_, LCP, and LSP of inverted leaves were reduced by 59.1%, 51.2%, 49.1%, 16.5%, and 13.1%, respectively, compared to normal leaves. After the inverted leaves recovered, the P_*Nmax*_, AQY, R_*D*_, LCP, and LSP of the leaves were reduced by 33.2%, 25.2%, 19.4%, 1.9%, and 16.0%, respectively, compared to the normal leaves.

**TABLE 2:** Photosynthetic light-response parameters of inverted leaves in 2021.

Times	Treatment	P_*Nmax*_ [μmol(CO_2_) m^–2^ s^–1^]	R_*D*_ [μmol(CO_2_) m^–2^ s^–1^]	AQY [mol(CO_2_) mol^–1^(photon)]	LCP [μmol(CO_2_) m^–2^ s^–1^]	LSP [μmol(CO_2_) m^–2^ s^–1^]
Treatments	CK	31.7 ± 1.2^a^	6.7 ± 0.2^a^	0.057 ± 0.001^a^	130.1 ± 1.3^a^	664.7 ± 12.1^a^
	Leaf inversion	13.0 ± 1.5^b^	3.3 ± 0.5^b^	0.029 ± 0.002_b_	108.7 ± 1.9^b^	577.3 ± 11.4^b^
Recovery	CK	17.4 ± 0.6*^a^*	5.9 ± 0.4^a^	0.036 ± 0.001^a^	130.5 ± 1.4^a^	629.6 ± 13.4^a^
	Leaf inversion	11.6 ± 1.1^b^	4.4 ± 0.3^b^	0.029 ± 0.001^b^	128.0 ± 2.1^a^	528.4 ± 15.3^b^

*P_Nmax_, the maximum net photosynthetic rate; R_D_, dark respiration rate; AQY, apparent quantum yield; LCP, light compensation point; LSP, light saturation point. Different letters indicate a statistically significant level at P < 0.05. Bars mean SE (n = 3).*

### Chlorophyll *a* Fluorescence Rise

**FIGURE 3:**
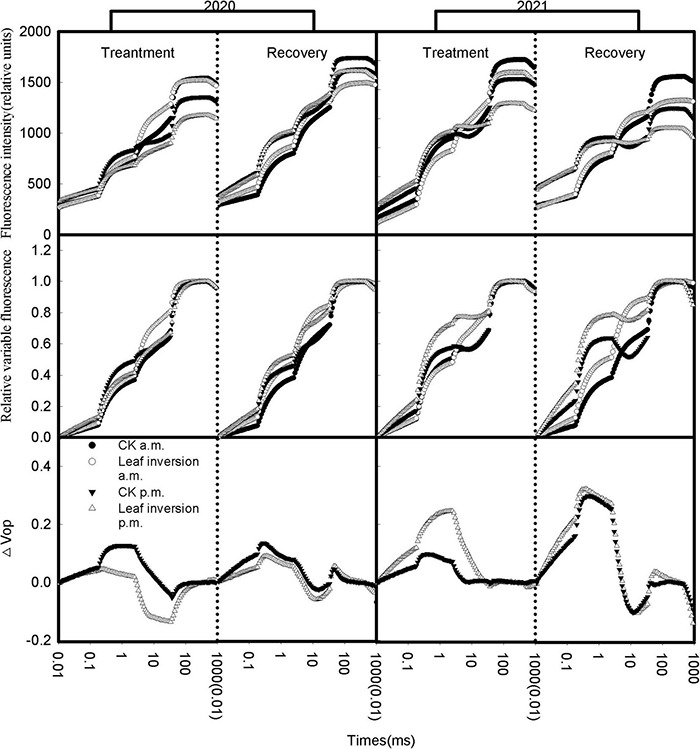
Native fluorescence induction curves and double O–P normalized OJIP transients at 10 and 15 (recovery) days after soybean leaf inversion. Each curve presents the average kinetics of five repetitions. Chlorophyll *a* fluorescence transient curves normalized between F_*O*_ and F_*P*_ expressed as V_*OP*_ [V_*OP*_ = (F_*t*_–F_*O*_)/(F_*P*_–F_*O*_)], △V_*OP*_ = V_*OP*_(treatment at 4 p.m.) – V_*OP*_(measurement at 9 a.m.). The vertical dashed line separates treatment from recovery. The values in parentheses are the starting values for the graph on the right.

When the chlorophyll *a* fluorescence induction curves were plotted on the logarithmic timescale as the horizontal coordinate and the immediate chlorophyll *a* fluorescence intensity of all treated leaves as the vertical coordinate, a rapid rise in the OJIP fluorescence transient was evident (Figure 3). Both the measurement at 4 p.m. and the leaf inversion resulted in a change in the shape of the chlorophyll *a* fluorescence induction curve, and when the inverted leaves were restored, the shape of the chlorophyll *a* fluorescence induction curve did not change. To further evaluate the changes in leaf photosynthetic performance under heat and high light at noon, a relative variable fluorescence curve [V_*OP*_ = (F_*t*_−F_*O*_)/(F_*m*_−F_*O*_)] was constructed to compare the differences in plant photosynthetic performance between 4 p.m. and 9 a.m. The value of each difference curve was the relative variable fluorescence value recorded at 4 p.m. minus the relative variable fluorescence value recorded at 9 a.m. [ΔV_*OP*_ = V_*OP*_(measurement at 4 p.m.)-V_*OP*_(measurement at 9 a.m.)]. The shape of the relative variable fluorescence curve of leaves recorded at 4 p.m. differed from that recorded at 9 a.m. In all treatments, changes in fluorescence transient curve shape caused by elevated temperature and high light could be clearly visualized by difference curves. In 2020, the difference curves for inverted leaves have a larger magnitude of variation compared to normal leaves, while in 2021 the difference curves for normal leaves have a larger magnitude of variation.

### Normalization of Chlorophyll *a* Fluorescence Transient Curves

**FIGURE 4:**
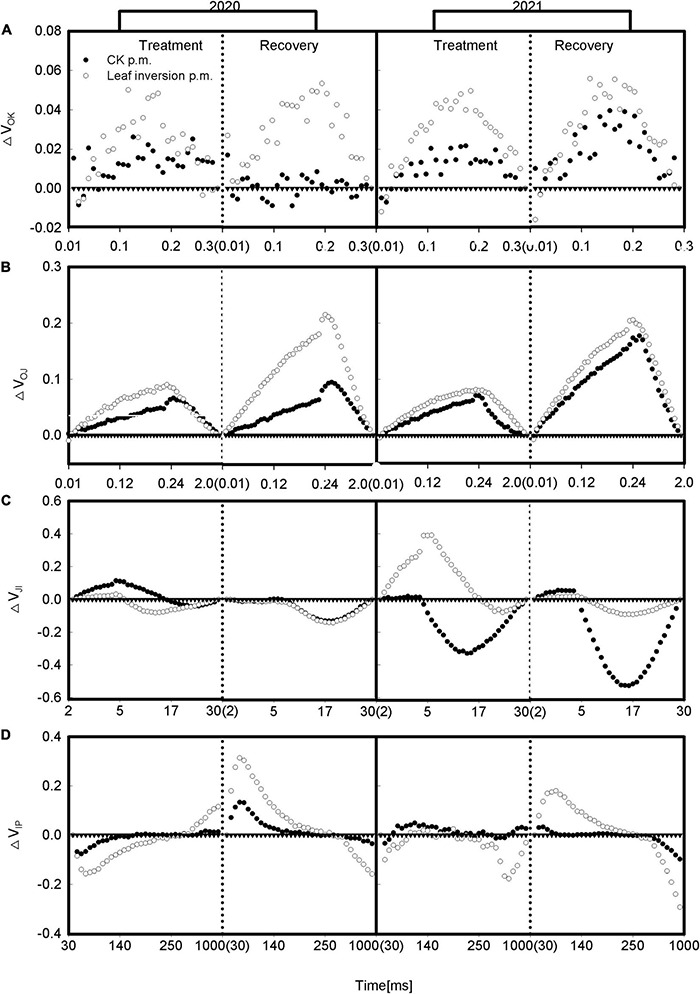
**(A–D)** Double normalization between F_*O*_ and F_*K*_ expressed as V_*OK*_ [V_*OK*_ = (F_*t*_–F_*O*_)/(F_*K*_–F_*O*_)], between F_*O*_ and F_*J*_ expressed as V_*OJ*_ [V_*OJ*_ = (F_*t*_–F_*O*_)/(F_*J*_–F_*O*_)], between F_*O*_ and F_*I*_ expressed as V_*OI*_ [V_*OI*_ = (F_*t*_–F_*O*_)/(F_*I*_–F_*O*_)], between F_*J*_ and F_*I*_ expressed as V_*JI*_ [V_*JI*_ = (F_*t*_–F_*J*_)/(F_*I*_–F_*J*_)], and between F_*I*_ and F_*P*_ expressed as V_*IP*_ [V_*IP*_ = (F_*t*_–F_*I*_)/(F_*P*_–F_*I*_)]. △V_*OX*_ = V_*OX*_(measurement at 4 p.m.)–V_*OX*_(measurement at 9 a.m.). Each curve presents the average kinetics of five repetitions. The vertical dotted line separates treatment from recovery. The values in parentheses are the starting values for the graph on the right.

To further elucidate the differences between treatments during the O-P phase of the chlorophyll *a* fluorescence transient, we, respectively, presented the differential curves for the main bands occurring during the O-P transient. The curves for these bands were constructed by subtracting the standardized fluorescence values of plants recorded at 4 p.m. (between O and K, O and J, J and I, or I and P, respectively) from the standardized fluorescence values of plants recorded at 9 a.m. (Figures 4A–D). The O-K normalized curve, called the L-band, provides information on the effective light absorption and energy utilization in the initial phase of photosynthesis. The leaves showed positive L-bands for all groups caused by elevated temperature and high light at 4 p.m. during treatment and recovery; the O-J normalized curve called K-band was used to check the status of the PSII donor side, where heat and high light at 4 p.m. caused all groups to show positive K-bands. The J-I normalized and I-P normalized curves indicate the balance between reduction and oxidation of the Q_*A*_ and plastoquinone (PQ) pools, respectively, and the J-I normalized curves showed a difference between years and treatments, with inverted leaves showing negative bands during treatment in 2020, while normal leaves showed positive bands and inverted leaves still showed negative bands after recovery, 2021 and 2020 were exactly the opposite, with leaves showing the highest negative bands during treatment and recovery. The I-P normalization curves differed between treatments, with inverted leaves showing a negative band during treatment and a positive band during recovery.

### Specific Fluxes per Reaction Center and Flux Ratios

**FIGURE 5:**
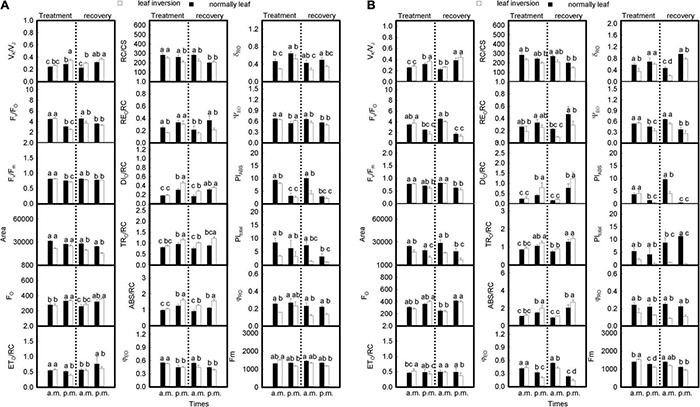
The JIP-test parameters of soybean leaf inversion (10 days) and recovery (15 days) evaluated during 2020 **(A)** and 2021 **(B)** at 9 a.m. and 4 p.m.; L, the combined stress of elevated temperature and high light; T, leaf inversion; F_*v*_/F_*m*_, maximum quantum yield of photosystem II (PSII); ABS/RC, absorption flux per reaction center (RC) at *t* = 0; DI_*O*_/RC, Dissipated energy flux per RC at *t* = 0; TR_*O*_/RC, Trapped energy flux per RC at *t* = 0; ET_*O*_/RC, Electron transport flux per RC at *t* = 0; Ψ_*EO*_, Probability that a trapped exciton moves an electron into the trapped electron transport chain beyond Q_*A*_^–^; φ_*EO*_, Quantum yield for electron transport at *t* = 0; δ_*RO*_, Efficiency with which an electron can move from the reduced intersystem electron acceptors to the photosystem I (PSI) end electron acceptors; φ_*RO*_, Quantum yield for the reduction of end acceptors of PSI per photon absorbed; V_*K*_/V_*J*_, Limitation/inactivation and possible damage of the oxygen-evolving complex; F_*V*_/F_*O*_, maximum ratio of quantum yields of photochemical and concurrent non-photochemical processes in PSII; Area, Density area over the chlorophyll *a* fluorescence transient delimited by a horizontal line at F_*m*_; F_*O*_, Minimum fluorescence, when all PSII RCs were open; F_*m*_, Maximum fluorescence, when all PSII RCs were closed; RC/CS, Density of active RCs (Q_*A*_ reducing RCs) per cross-section at *t* = 0; PI_ABS_, performance index on absorption basis; PI_total_, efficiency of energy conservation from absorbed photons to the reduction of PSI end acceptors. Data are expressed as means ± SEs (*n* = 5). Different letters represent significant differences (*P* < 0.05) between treatment and time of measurement. The vertical dotted line separates the treatment from the recovery.

During the leaf treatment (Figures 5A,B and Table 3), the limitation/inactivation and possible damage of the oxygen-evolving complex (OEC) (V_*K*_/V_*J*_), RE_*O*_/RC, dissipated energy flux per RC at *t* = 0 (DI_*O*_/RC), trapped energy flux per RC at *t* = 0 (TR_*O*_/RC), absorption flux per RC (ABS/RC), efficiency with which an electron can move from the reduced intersystem electron acceptors to the photosystem I (PSI) end electron acceptors (δ_*RO*_), and minimum fluorescence when all PSII RCs were open (F_*O*_) values increased at 4 p.m. for inverted and normal leaves compared to measurements at 9 a.m., while F_*V*_/F_*O*_, F_*v*_/F_*m*_, Area, electron transport flux per RC at *t* = 0 (ET_*O*_/RC), the density of active RCs (Q_*A*_ reducing RCs) per cross-section at *t* = 0 (RC/CS), quantum yield for electron transport at *t* = 0 (φ_*EO*_), the probability that a trapped exciton moves an electron into the trapped electron transport chain beyond Q_*A*_^–^ (Ψ_*EO*_), PI_total_, quantum yield for the reduction of end acceptors of PSI per photon absorbed (φ_*RO*_), and F_*m*_ values decreased at 4 p.m. When inverted leaves recovered, the trend in values was consistent with the leaves during treatment except for ET_*O*_/RC and PI_total_ values; compared to measurements at 9 a.m., ET_*O*_/RC values increased at 4 p.m. after inverted leaves recovered in 2020, but the difference was not significant, but significantly decreased in 2021; PI_total_ values decreased at 4 p.m. for all groups in 2020, but in 2021 normal leaves increased slightly at 4 p.m. Leaf V_*K*_/V_*J*_, RC/CS, RE_*O*_/RC, DI_*O*_/RC, ABS/RC, δ_*RO*_, PI_ABS_, and PI_total_ values differed significantly (*P* < 0.05) between measurements at 9 a.m. and 4 p.m. during treatment, and their values also showed significant differences (*P* < 0.05) between inverted and normal leaves; F_*V*_/F_*O*_, F_*O*_, F_*m*_, and ET_*O*_/RC values were significantly different between 9 a.m. and 4 p.m. measurements (*P* < 0.05), while leaf inversion did not affect their values significantly (*P* > 0.05); φ_*RO*_ values were not significantly different between 9 a.m. and 4 p.m. measurements (*P* > 0.05), but leaf inversion affected their values significantly (*P* < 0.05); F_*v*_/F_*m*_, TR_*O*_/RC, φ_*EO*_, and Ψ_*EO*_ values were significantly different between 9 a.m. and 4 p.m. measurements (*P* < 0.05); F_*v*_/F_*m*_, TR_*O*_/RC, φ_*EO*_, and Ψ_*EO*_ values were significantly different between 9 a.m. and 4 p.m. measurements. F_*v*_/F_*m*_, TR_*O*_/RC, φ_*EO*_, and Ψ_*EO*_ values were significantly different between 9 a.m. and 4 p.m. measurements (*P* < 0.05), and leaf inversion had no effect or reached a significant level. There was no significant difference in the Area value measured at 9 a.m. and 4 p.m. in 2020 (*P* > 0.05), and leaf inversion had no effect on its value, but there was a significant difference between the treatment groups in 2021 (*P* < 0.05). PI_ABS_ and PI_total_ were significantly lower (*P* < 0.05) for the 4 p.m. measurement compared to the 9 a.m. measurement. The interaction of leaf inversion and time of measurement on chlorophyll *a* fluorescence parameters in both years was not significant only for V_*K*_/V_*J*_, ET_*O*_/RC, RE_*O*_/RC, PI_ABS_, F_*O*_, TR_*O*_/RC, RC/CS, δ_*RO*_, and φ_*RO*_.

**TABLE 3:** ANOVA of effects of elevated temperature and high light stress on fluorescence parameters.

2020																			
**Treatments**	**Source of variation**	**V_*K*_/V_*J*_**	**F_*V*_/F_*O*_**	**F_*v*_/F_*m*_**	**Area**	**F_*O*_**	**ET_*O*_/RC**	**RC/CS**	**RE_*O*_/RC**	**DI_*O*_/RC**	**TR_*O*_/RC**	**ABS/RC**	**φ_*EO*_**	**δ_*RO*_**	****Ψ**_*EO*_**	**PI_ABS_**	**PI_total_**	**φ_*RO*_**	**F_*m*_**
Leaf version	L	**	*	**	ns	*	*	*	**	**	**	**	**	**	**	**	ns	ns	*
	T	**	ns	*	ns	ns	ns	**	*	**	**	**	ns	*	ns	**	**	*	ns
	L×T	ns	*	**	ns	ns	ns	ns	ns	**	ns	*	ns	ns	*	ns	ns	ns	*
Recovery	L	**	*	*	*	**	**	ns	*	**	*	**	*	ns	*	**	**	ns	*
	T	**	ns	*	ns	ns	*	**	*	**	**	**	*	**	*	*	**	**	*
	L×T	ns	ns	ns	**	ns	ns	ns	ns	ns	ns	ns	ns	ns	ns	*	*	ns	ns

**2021**																			

Leaf version	L	**	**	**	**	*	*	*	*	**	**	**	**	**	**	**	*	ns	**
	T	*	ns	ns	**	ns	ns	*	*	**	ns	*	*	**	*	*	**	**	ns
	L×T	ns	ns	ns	ns	ns	ns	ns	ns	**	ns	ns	**	ns	**	ns	**	ns	**
Recovery	L	**	**	**	**	**	*	*	**	**	**	**	**	**	**	**	*	ns	*
	T	*	*	ns	**	ns	**	*	**	*	ns	*	**	**	*	**	**	**	ns
	L×T	ns	ns	ns	ns	ns	ns	ns	ns	ns	ns	ns	ns	ns	ns	**	ns	ns	ns

L, Light treatment; T, temperature treatment. ** significantly different at P < 0.01; * significantly different at P < 0.05; ns, the difference was not significant.

After leaf inversion recovery (Figures 5A,B and Table 3), leaf V_*K*_/V_*J*_, ET_*O*_/RC, RE_*O*_/RC, DI_*O*_/RC, ABS/RC, φ_*EO*_, Ψ_*EO*_ PI_ABS_, and PI_total_ values differed significantly (*P* < 0.05) between the 9 a.m. and 4 p.m. measurements, and their values also showed significant differences (*P* < 0.05) between inverted and normal leaves; F_*V*_/F_*O*_, F_*O*_, F_*m*_, F_*v*_/F_*m*_, Area, and TR_*O*_/RC values differed significantly (*P* < 0.05) between the 9 a.m. and 4 p.m. measurements, while the effect of leaf inversion on their values varied from year to year; RC/CS, δ_*RO*_, and φ_*RO*_ values differed significantly (*P* < 0.05) between the 9 a.m. and 4 p.m. measurements, and the effect of leaf inversion on their values varied from year to year. The interaction of leaf inversion and time of measurement on other chlorophyll *a* fluorescence parameters was not significant in both years except for Area, PI_ABS_, and PI_total_ values.

### Principal Component Analysis

**FIGURE 6:**
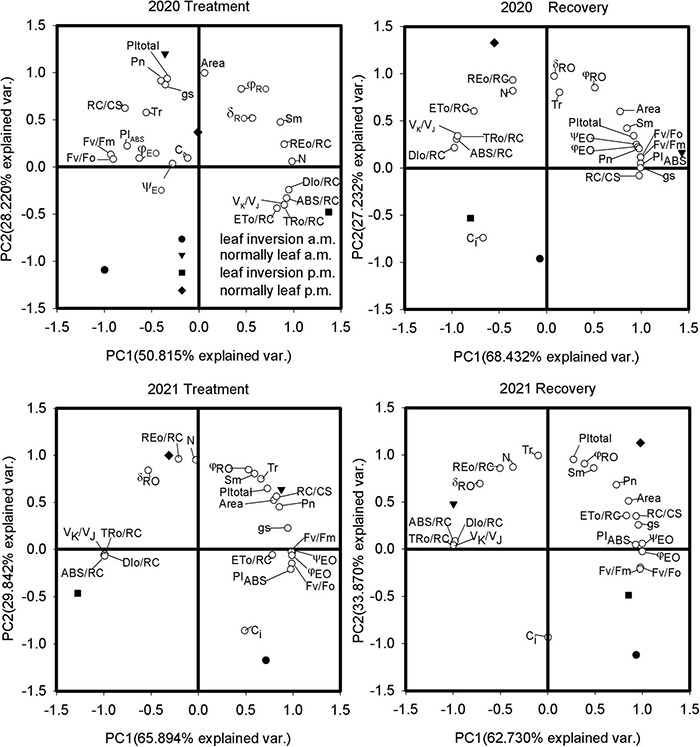
Principal component analysis of variability of JIP-test and gas exchange parameters of soybean leaves for 10 and 15 days (recovery) after leaf inversion in 2020 and 2021.

The PCA of the fluorescence and gas exchange parameters of the examined soybean cultivars revealed both differences and similarities between the different treatments (Table 4 and Figure 6), with the first 2 principal components (PCs) accounting for 79.0%–96.6% of the total variance. Leaves were treated with the first PC (PC1) reflecting 50.8% and 65.9% of the total variance in 2020 and 2021, respectively, and the second PC (PC2) reflecting 28.2% and 29.8% of the total variance, respectively. The results of PCA indicated that normal leaves measured at 9 a.m. exhibited higher photosynthesis (P_*n*_), performance indexes (PI_total_ and PI_ABS_), and number of RCs (RC/CS); inverted leaves measured at 4 p.m. were characterized by higher specific activity parameters (ABS/RC, DI_*O*_/RC, and TR_*O*_/RC), while normal leaves had higher loads on Area, δ_*RO*_, and RE_*O*_/RC. After inverted leaf recovery, the PC1 reflected 68.4% and 62.7% of the total variation in 2020 and 2021, respectively, and the PC2 reflected 27.2% and 33.9% of the total variation, respectively. Normal leaves measured at 9 a.m. in 2020 and 4 p.m. in 2021 exhibited higher P_*n*_, performance indexes (PI_total_ and PI_ABS_), and number of RCs (RC/CS). P_*n*_ was positively correlated with PI_ABS_, PI_total_, and RC/CS and negatively correlated with ABS/RC, DI_*O*_/RC, and TR_*O*_/RC.

**TABLE 4:** The results of principal component analysis (PCA).

Year		2020				2021			
Different measures		Treatments		Recovery		Treatments		Recovery	
Principle factors		PC1	PC2	PC1	PC2	PC1	PC2	PC1	PC2
Eigen vector	RC/CS	–0.782	0.623	**0.978**	–0.080	0.796	0.524	**0.935**	0.349
	V_*K*_/V_*J*_	**0.901**	–0.403	−**0.940**	0.340	−**0.991**	–0.070	−**0.997**	0.040
	Area	0.058	**0.997**	0.777	0.600	0.825	0.565	0.854	0.511
	Fv/Fm	−**0.933**	0.133	**0.992**	0.116	**0.990**	–0.034	**0.979**	–0.196
	Fv/Fo	−**0.907**	0.083	**0.993**	0.115	**0.987**	–0.150	**0.975**	–0.213
	Sm	0.860	0.476	0.849	0.422	0.592	0.804	0.486	0.860
	N	**0.983**	0.059	–0.358	0.819	–0.026	**0.952**	–0.367	0.870
	ABS/RC	**0.926**	–0.330	−**0.952**	0.307	−**0.998**	–0.058	−**0.989**	0.066
	DIo/RC	**0.949**	–0.242	−**0.975**	0.216	−**0.990**	–0.050	−**0.980**	0.086
	TRo/RC	**0.901**	–0.402	−**0.940**	0.340	−**0.991**	–0.069	−**0.997**	0.040
	ETo/RC	0.824	–0.438	–0.768	0.604	0.781	–0.058	0.831	0.355
	(REo)/RC	**0.901**	0.240	–0.358	**0.934**	–0.209	**0.962**	–0.505	0.858
	ΨEo	–0.280	0.036	**0.948**	0.249	**0.984**	–0.027	**0.998**	0.058
	φEo	–0.632	0.095	**0.963**	0.222	**0.987**	–0.062	**0.999**	–0.027
	δRo	0.563	0.518	0.079	**0.975**	–0.531	0.841	–0.717	0.695
	φRo	0.447	0.828	0.508	0.853	0.530	0.848	0.388	**0.906**
	PI_ABS_	–0.759	0.224	**0.994**	0.048	**0.976**	–0.211	**0.929**	0.046
	PI_total_	–0.336	**0.937**	**0.921**	0.342	0.728	0.648	0.271	**0.951**
	Tr	–0.558	0.577	0.136	0.802	0.660	0.750	–0.100	**0.994**
	gs	–0.356	0.872	**0.991**	0.003	**0.944**	0.225	**0.957**	0.255
	P_*n*_	–0.402	**0.915**	**0.977**	0.206	0.855	0.453	0.725	0.683
	C_*i*_	–0.120	0.094	–0.671	–0.740	0.489	–0.860	0.004	−**0.936**
Eigenvalues		13.283	5.982	15.139	5.907	14.976	6.086	13.931	7.321
Variation explained (%)		50.815	28.220	68.432	27.232	65.894	29.842	62.730	33.870
Cumulative proportion (%)		50.815	79.035	68.432	95.665	65.894	95.737	65.894	96.600

*Vector loadings ≥ 0.90 are mentioned in bold.*

## Discussion

Under natural conditions, a combination of high light and heat stress in summer is the main environmental stress that leads to a decrease in plant photosynthesis, usually starting with a reduction in the production of photosynthetic assimilates under mild abiotic stress when leaves avoid water dissipation by reducing stomatal aperture and thus limiting the mesophyll conductance to CO_2_, at which point diffusive limitation is the main cause of the decrease in leaf photosynthetic capacity (Das et al., 2015; Bahamonde et al., 2018; Fanourakis et al., 2019; Mihaljević et al., 2020). As the stress level increased and time increased, the leaf photosynthetic apparatus was damaged and PSII photochemical activity was reduced in addition to diffusive limitation, and at this time, the nondiffusion limitation was better than diffusion limitation (Hazrati et al., 2016; Jiang et al., 2021). In this study, the P_*n*_ and gs of inverted leaves and normal leaves were significantly reduced at 4 p.m. during the treatment, and C_*i*_ of normal leaves was also significantly reduced at noon, but C_*i*_ in inverted leaves behaved differently in both years, with no significant difference between 9 a.m. and 4 p.m. C_*i*_ in inverted leaves in 2020, while the difference reached a significant level in 2021, which could be caused by climatic factors between years but could at least suggest that the reduction in P_*n*_ of normal leaves can be explained by a reduction in gs (Pandey et al., 2007) and that inverted leaves may be more susceptible to photooxidative damage and damage to the photosynthetic apparatus at elevated temperature and high light compared to normal leaves, which is supported by the decrease in F_*v*_/F_*m*_ values and performance indexes (PI_ABS_ and PI_total_) and the increase in DI_*O*_/RC values in inverted leaves at noon. Between the 2 years, the T_*r*_ values of normal leaves were slightly elevated or flat in the noon compared to the morning measurement, but T_*r*_ values of inverted leaves were significantly lower in the noon, which may also be one of the reasons for the lower P_*n*_ of inverted leaves compared to normal leaves, as the lower transpiration rate leads to higher leaf temperature, and the photoinhibition of photosynthesis depends on Temperature, the increase of leaf temperature will strengthen the photoinhibition, thereby greatly reducing the photosynthetic capacity of inverted leaves (Avola et al., 2008; Das et al., 2015; Gago et al., 2015; Durand et al., 2020). After the recovery of inverted leaves, the C_*i*_ values of normal and inverted leaves did not decrease with the decrease of P_*n*_ and gs values under elevated temperature and high light, and the C_*i*_ values of inverted leaves were higher than those of normal leaves, indicating that the irreversible damage to the photosynthetic apparatus of inverted leaves occurred under heat and high light (Mihaljević et al., 2020; Cohen et al., 2021), but the factor of shorter recovery time of inverted leaves could not be ignored. To obtain the adaptation of inverted leaves to light under the combined stress of high light and elevated temperature, the response of P_*n*_ to several light intensities of PPFD was evaluated. In this study, the P_*Nmax*_, AQY, R_*D*_, LCP, and LSP values of inverted leaves were lower than those of normal leaves under treatment and recovery conditions, indicating that the photosynthetic potential of leaves under elevated temperature and high light was reduced and the ability to utilize strong light was weakened in inverted leaves, similar to the studies by Proietti and Palliotti (1997), Xu et al. (2013), and Paradiso et al. (2020). It is worth noting that the P_*Nmax*_ of normal leaves decreases significantly from treatment to recovery, and this may be due to the fact that soybeans are susceptible to high temperature stress during the filling stage, which induced and accelerated the senescence of inverted leaves in advance (i.e., PI_*total*_ of inverted leaves was significantly lower than that of normal leaves under elevated temperature and high light), while the photosynthetic products of normal leaves efficiently transport to the reproductive organs during the recovery phase, accelerating leaf senescence, resulting in that the P_*Nmax*_ of the normal leaves was significantly lower during the recovery.

Many studies have assessed the harmful effects of heat on photosynthesis, where elevated temperatures reduce CO_2_ fixation by inhibiting photosynthetic system activity. The inactivation of PSII in plants under complex stress in summer leads to a decrease in photosynthetic capacity (Fanourakis et al., 2019; Jiang et al., 2021). Some researchers have noted that F_*v*_/F_*m*_ values of normal leaves range from 0.75 to 0.83 and that a decrease in this parameter indicates that PSII has been damaged (Krause and Weis, 1991). According to a certain one, an increase in F_*O*_ is one of the signs of photoinhibition (Uhrmacher et al., 1995). It has also been suggested that an increase in DI_*O*_/RC and a decrease in Ψ_*EO*_ can be prepared to identify photoinhibition rather than F_*v*_/F_*m*_ (Jiang et al., 2008). In this study, the F_*v*_/F_*m*_ values of normal and inverted leaves were significantly lower at 4 p.m. compared to morning measurements, and the decrease was higher in inverted leaves than that of normal leaves. The F_*v*_/F_*m*_ values ranged from 0.62 to 0.71 at 4 p.m. during treatment for inverted leaves, while normal leaves ranged from 0.71 to 0.75; after recovery of inverted leaves, the F_*v*_/F_*m*_ of the inverted leaves in 2021 decreased significantly at 4 p.m. (F_*v*_/F_*m*_ = 0.55) and that of the normal leaves is 0.61, At the same time, compared with the morning measurement, the F_*v*_/F_*m*_ and Ψ_*EO*_ values of the inverted leaves decreased at 4 p.m., and the increase in DI_*O*_/RC and F_*O*_ values were higher than those of the normal leaves. The LSP of the inverted leaf is between 528.4 and 577.3 μmol(CO_2_) m^–2^ s^–1^, while the normal leaf is between 629.6 and 664.7 mol(CO_2_) m^–2^ s^–1^, and the temperature at 4 p.m. in summer is 38.2°C, and the light intensity is 1,512 μmol(CO_2_) m^–2^ s^–1^, which is much higher than its light saturation point, which causes the light system to be overexcited. All the above mentioned results indicate that the combined stress of elevated temperature and high light in summer promotes PSII inhibition of inverted leaves and stronger energy dissipation. This is supported by the significant increase in DI_*O*_/RC value at 4 p.m. and the PCA results. This is similar to the study by Mihaljević et al. (2020) on apples.

In recent years, there has been considerable interest in using chlorophyll *a* fluorescence and related parameters to assess the effects of abiotic stress on the photosynthetic structure, using chlorophyll *a* fluorescence kinetics to characterize plant tolerance to abiotic stresses at the PSII level (Hussain et al., 2019; Rastogi et al., 2020). In fact, elevated temperature and high light stress result in significant changes in the shape of the chlorophyll fluorescence induction curve (Mihaljević et al., 2020). In this study, the combined high light and elevated temperature stresses significantly affected PSII performance during treatment and recovery of leaves, and these effects were visible in the variable fluorescence curves and relative variable fluorescence curves during treatment and recovery (Figures 3, 4). The typical shape of the OJIP transient curve has 3 main phases, namely, O-J, J-I, and I-P (Strasser and Srivastava, 1995). The J-I phase reflects the reduction of electron carriers (plastoquinone and plastocyanin) between PSII and PSI (Tóth et al., 2007). In this study, the increase in J-I transients in normal and inverted leaves differed by year, and an increase in J-I transients was observed in inverted leaves compared to normal leaves, which supports the vulnerability of the electron carriers of the inverted leaves to elevated temperature and high light and the partial reduction of the PQ pools between PSII to PSI, which is consistent with the reduction in the PQ pools under elevated temperature reported by Mihaljević et al. (2020). The I-P phase is the slowest fluorescence rise and is associated with a decrease in electron transport proteins (Ceppi et al., 2012). Previous studies have confirmed that I-P is relatively sensitive to various abiotic stress (Schansker et al., 2005). In this study, inverted leaves showed a significant positive peak change during recovery, suggesting that damage to the PSI structure, loss of function in inverted leaves, and electron transport on the PSI receptor side may have been inhibited.

The PSII and OEC are one of the main stress-sensitive sites in the photosynthetic apparatus (Adamski et al., 2011). Previous studies have shown that high light and elevated temperature reduce PSII activity and electron transfer efficiency (Boguszewska-Mańkowska et al., 2018). In this study, the active RC of normal and inverted leaves was significantly reduced at 4 p.m. (supported by the increase in ABS/RC). The increase in ABS/RC may be due to the increase in antenna size or partial PSII RC inactivation, which can be confirmed by a decrease in active RC per excitation cross-section (RC/CS). The inactivation of RC is considered to be a photoprotective mechanism, because part of the RC is transformed into a so-called “heat sink” by Stefanov et al. (2011), to dissipate excess excitation energy to prevent excessive excitation of PSII. The significant increase in dissipated energy (DI_*O*_/RC) supports the change of RC function. Compared with the morning measurement, the TR_*O*_/RC and DI_*O*_/RC of the inverted leaves increased by 31.5% and 135.1%, respectively, at 4 p.m., while the normal leaves increased by 21.4% and 39.1%, respectively. These findings suggest that inverted leaves dissipate excitation energy in the form of more heat and fluorescence and more severe photoinhibition, which is consistent with the research results of Mihaljević et al. (2020). ET_*O*_/RC describes the electron transport flux of each RC, which reflects the activity of active RCs. The ET_*O*_/RC values decreased at 4 p.m., but some researchers have shown that ET_*O*_/RC remained constant at heat and high light or that the ET_*O*_/RC values increased at elevated temperatures (Faria-Silva et al., 2019; Mihaljević et al., 2020). In this study, the ET_*O*_/RC values of inverted leaves decreased significantly at 4 p.m. compared with 9 a.m., while normal leaves remained essentially unchanged, which further supports the hypothesis that some of the RC is converted into a “heat sink” and also indicates that the activity of active RC in inverted leaves is reduced under high light and elevated temperature, which is consistent with the study by Mihaljević et al. (2020). Elevated temperature and high light also affect both the donor and acceptor sides of PSII (Buchner et al., 2015): on the donor side, OEC inactivated, as shown in this study by a significant increase in the positive K-band at 300 ms and V_*K*_/V_*J*_, which may be due to the loss of manganese cluster function in PSII at elevated temperature, resulting in an imbalance electron transport between the OEC and the PSII RC; while at the acceptor site, the electron transport between Q_*A*_^–^ and Q_*B*_^–^ is inhibited (Gu et al., 2017), and the disruption of the electron transport chain is due to the dissociation of the LHCII from PSII, which helps to avoid excessive reduction of PQ and protect PSII from damage (Galić et al., 2020). These findings can be supported by the significant reduction in the Area value of inverted leaves at 4 p.m. (because Area refers to the free PQ pool). A positive L-band indicates a lower energy connection (Kalaji et al., 2014; Jiang et al., 2021). Both inverted and normal leaves exhibited significant positive L- and K-bands at elevated temperature and high light, but the positive L- and K-bands were more pronounced in inverted leaves than in normal leaves, indicating more severe OEC damage. Elevated temperature and high light not only affect the function of PSII but also adversely affect the electron flow on the PSI acceptor side (φ_*RO*_, R_*EO*_/RC, and δ_*RO*_) (Kalaji et al., 2014). A large number of studies have shown that δ_*RO*_ values increase in plants after heat stress (Oukarroum et al., 2009; Mihaljević et al., 2020). The same, but inverted leaves exhibit higher R_*EO*_/RC and δ_*RO*_ than normal leaves, indicating that the reduction in electron transport efficiency from the intersystem electron carrier to the PSI receptor side of the inverted leaves is even greater. High light and elevated temperature did not appear to have an effect on φ_*RO*_ values, as ANOVA showed no significant differences between treatments; however, inverted leaves had significantly lower φ_*RO*_ compared with normal leaves, which may be due to a reduction in PSI content in inverted leaves (Yan et al., 2013). In this study, F_*V*_/F_*O*_ was significantly lower in inverted and normal leaves under high light and elevated temperature, but the decrease was higher in inverted leaves than in normal leaves, indicating that electron transport was impaired during photosynthesis in inverted leaves compared to normal leaves, which is similar to the results of Janka et al. (2020) who treated microalgae (*Tetradesmus wisconsinensis*) with bicarbonate.

The overall photosynthetic performance of both normal and inverted leaves was reduced under elevated temperature and high light complex stress conditions, which can be explained by a decrease in the performance indexes (PI_ABS_ and PI_total_). The expression of performance index PI_ABS_ is the product of three independent characteristics: the density of active RC per PSII antenna chlorophyll (RC/ABS), the maximum quantum efficiency of PSII (F_*v*_/F_*m*_), and the electron transport beyond Q_*A*_ (Ψ_*EO*_) (Strasser et al., 2000; Kalaji et al., 2016). In this study, the decrease in PI_ABS_ appeared to be associated with a decrease in RC/ABS and Ψ_*EO*_ values, as the decrease in their values is greatest at elevated temperatures and high light. The PI_ABS_ values of normal and inverted leaves decreased by 63.2%–66% and 68%–90% at 4 p.m., respectively, compared to 9 a.m. Compared with PI_ABS_, the PI_total_ values not only reflect PSII photosynthetic electron transfer activity but also relate to changes in PSI-related processes (Kalaji et al., 2014; Jiang et al., 2021). Therefore, some researchers suggest that PI_total_ is more sensitive to abiotic stresses than PI_ABS_. Some researchers also concluded that the sensitivity of PI_ABS_ and PI_total_ to abiotic stress varied depending on environmental factors (Mallick and Mohn, 2003; Mihaljević et al., 2020). In this study, the PI_total_ values of normal and inverted leaves decreased by 24.1%–25.9% and 5.9%–66.7%, respectively, at 4 p.m. compared to 9 a.m. This is consistent with the trend in P_*N*_ values. The decrease in PI_total_ may be related to the loss of PSII activity, leading to a reduction in the electron transport chain and disruption of PSI function (Kalaji et al., 2014; Oukarroum et al., 2018). In this study, the PI_ABS_ values of inverted leaves appeared to be more sensitive to elevated temperature and high light compared with PI_total_. The fact that PI_ABS_ and PI_total_ were still not restored to the levels of normal leaves after the recovery of inverted leaves may be due to the higher degree of damage suffered by inverted leaves under elevated temperature and high light, and the effective repair capacity of the photosynthetic apparatus (synthesis of new proteins to replace damaged core proteins) of inverted leaves remained lower than the photooxidative damage capacity after recovery.

## Conclusion

Stress resistance is a very important factor for the successful production of soybean in the increasingly demanding agroecological conditions. Our data indicate that a more severe photooxidative damage occurs in inverted leaves under high light and elevated temperature conditions compared to normal leaves, as evidenced by OEC inactivation, inhibition of electron transport, and inactivation of some PSII RCs. High light and elevated temperature significantly reduced the performance indexes (PI_ABS_ and PI_total_) of inverted leaves. The PI_ABS_ values of inverted leaves were more sensitive to elevated temperature and high light. The inhibition of electron transport and the inactivation of PSII RCs in inverted leaves under combined high light and elevated temperature stresses were responsible for the significant reduction in P_*n*_. Due to climate change, in the future, it will be required a better understanding of interactions between soybean and their environment to achieve better adaptability and thus the productivity of future cultivars.

## Data Availability Statement

The raw data supporting the conclusions of this article will be made available by the authors, without undue reservation.

## Author Contributions

CW and JZ designed the research. CW, JR, QG, LZ, and CL performed the research. CW, CL, and JZ analyzed the data. CW, LZ, and JZ wrote the manuscript. All authors contributed to the article and approved the submitted version.

## Conflict of Interest

The authors declare that the research was conducted in the absence of any commercial or financial relationships that could be construed as a potential conflict of interest.

## Publisher’s Note

All claims expressed in this article are solely those of the authors and do not necessarily represent those of their affiliated organizations, or those of the publisher, the editors and the reviewers. Any product that may be evaluated in this article, or claim that may be made by its manufacturer, is not guaranteed or endorsed by the publisher.
